# How nursing students’ risk perception affected their professional commitment during the COVID-19 pandemic: the mediating effects of negative emotions and moderating effects of psychological capital

**DOI:** 10.1057/s41599-023-01719-6

**Published:** 2023-05-04

**Authors:** Junlong Li, Changping Huang, Yili Yang, Jie Liu, Xiaojun Lin, Jay Pan

**Affiliations:** 1 HEOA Group, West China School of Public Health and West China Fourth Hospital, Sichuan University, Chengdu, Sichuan China; 2 Sichuan Vocational College of Health and Rehabilitation, Zigong, Sichuan China; 3 School of Public Administration, Sichuan University, Chengdu, Sichuan China

**Keywords:** Health humanities, Medical humanities, Psychology

## Abstract

Nurses play a pivotal role in the delivery of medical services. Professional commitment is crucial for nursing professionals’ long-term, healthy, and sustainable development. However, nursing students’ professional commitment levels are currently unsatisfactory in China, especially given that the COVID-19 pandemic has posed unprecedented challenges to the profession. Therefore, studies investigating the professional commitment levels of nursing students and the underlying influencing factors are urgently required. This study explored how nursing students’ risk perceptions, negative emotions, and psychological capital affected their professional commitment during the COVID-19 pandemic. A cross-sectional study was conducted among nursing students using risk perception, professional commitment, negative emotions, and psychological capital scales. An analysis of 1142 Chinese nursing students suggested that nursing students’ risk perception positively impacted professional commitment and that negative emotions mediated this association. Importantly, psychological capital moderates the mediating effect of negative emotions and can buffer the negative emotions caused by risk perception. This study demonstrated that effective intervention strategies should be implemented in multiple dimensions such as education, individual, public and society to improve the professional commitment of nursing students.

## Introduction

Nursing professionals play a pivotal role in the delivery of healthcare services. Professional commitment has been recognized as a significant contributor to the healthy, stable, and sustainable development of nursing professionals (Cheng et al., 2021). Professional commitment refers to the attitudes and behaviors of individuals regarding their occupations and the time and energy they would like to spend in their field of work (Lian et al., 2005). The professional commitment of nursing students largely reflects their degree of dedication and positive attitudes toward their occupation, which serve as powerful predictors of their future work commitment. As such, those with higher degrees of professional commitment during academic training will likely demonstrate better professional commitment after becoming registered nurses (Kong et al., 2016**;** Wang et al., 2023). The COVID-19 pandemic has created a challenging climate for healthcare professionals (Labrague and de Los Santos, 2021; Yang et al., 2020), under which the professional commitment of nursing professionals has been negatively impacted by prolonged work hours, increased workload, and pressure, along with a shortage of medical and protective facilities and interruption of social networks in their personal lives (Duran et al., 2021). Given that COVID-19 is still a global pandemic and the shortage of nursing professionals is a critical issue, the subsequent uncertainty of future nursing professionals should be urgently addressed (WHO, 2023; Xu and Wu, 2020). Under such circumstances, promoting nursing students’ professional commitment has become essential to minimize the loss of the nursing workforce potentially induced by the ongoing pandemic (Hua et al., 2022). Therefore, there is an urgent need to investigate nursing students’ levels of professional commitment and the underlying influencing factors to inform the implementation of effective intervention measures for the healthy, stable, and sustainable development of nursing talent, especially considering that Chinese nursing students present unsatisfactory professional commitment (Wang and Yu, 2021).

Previous studies have shown that risk perception is an important factor that affects professional commitment (Lv et al., 2020**;** Zeng et al., 2022). Although a connection between risk perception and professional commitment has been established in previous studies, evidence regarding how the former affects the latter remains poorly explored, not to mention providing insights into the current state of nursing candidates during major public health crises. As an essential part of professional training to be accomplished by nursing students before their registration, clinical internships have become a major factor resulting in dramatically increased risk perception during the COVID-19 pandemic, which might further compromise their dedication to their future careers as nurses (Kim et al., 2022). Negative emotions and psychological capital are frequently observed during major public health crises, largely affecting individuals’ behaviors and decision-making choices (Kheirallah et al., 2021; Meseguer de Pedro et al., 2021; Sun et al., 2021; Wang et al., 2021). Based on a review of the current literature, no study has examined the interaction mechanisms between the risk perception, negative emotions, psychological capital, and professional commitment of nursing students during the COVID-19 pandemic. Therefore, this study aimed to explore the potential connection mechanism between risk perception and professional commitment in Chinese nursing students during the COVID-19 pandemic and to investigate the role of negative emotions and psychological capital in the relationship between risk perception and professional commitment.

Owing to the global public health crisis, increased awareness has been raised toward risk perception as an inevitable issue during the pandemic (Alicea-Planas et al., 2021). Risk perception refers to individuals’ feelings, thoughts, judgments, and cognition of external things with objective risks. It emphasizes the impact on cognition caused by the experience obtained from individuals’ subjective feelings and intuitive judgments (Cori et al., 2020). Previous studies have reported that people experience a higher level of risk perception when approaching the epicenter of a crisis (Kasperson et al., 1988**;** Slovic, 1987). Nursing students tend to have much higher levels of risk perception during the pandemic, as they are exposed to real-world clinical settings while working on their clinical internships, and prolonged high-risk perception would likely cause undesired consequences from a long-term perspective **(**Karadas et al., 2022; Kim et al., 2022; Serrano-Gómez et al., 2022). According to the transactional model of stress and coping, individuals experience stress when surrounding situations or events are perceived as risky (Lazarus and Folkman, 1984; Lazarus, 2000). When risk-induced stress exceeds the self-control range, individuals choose behaviors such as estrangement and escape (Folkman and Lazarus, 1980). Based on this mechanism, it is not difficult to imagine that once increased stress is induced by a higher level of perceived risk of COVID-19, nursing students will hesitate to proceed with their academic training toward that risky occupation. Multiple studies have reported that nurses express an intention to quit their jobs because of their risk perception of COVID-19, which increases their fear (Liu et al., 2022). In this context, Hypothesis 1 was tested in our study: risk perception is negatively associated with the level of professional commitment in nursing students.

During major public health crises and social disasters, risk perception can affect individuals’ emotional responses, behavioral attitudes, and decision-making outcomes (Song et al., 2022). Previous studies indicate that risk perception positively correlates with negative emotions (An et al., 2022**;** Yin et al., 2021). Negative emotions are defined as the subjective feelings and emotional states of individuals who are depressed and continue to fall into unhappiness-activated states, such as depression, tension, anxiety, and stress (Lovibond and Lovibond, 1995). Many studies have shown that negative emotions tend to affect medical workers and college students during the COVID-19 pandemic (Han et al., 2022; Shreffler et al., 2020; Yin et al., 2021; Zhang, 2022). Nursing students exhibit negative emotions (Zou et al., 2020). Long-term negative emotions lead to the continuous depletion of individuals’ psychological resources, which results in a low psychological state (An et al., 2022). This is reflected in medical students’ learning motivations becoming increasingly negative over time, along with their weakened professional commitment, which might eventually lead to abandoning their occupation (Zhou and Dong, 2018). Therefore, Hypothesis 2 was proposed: negative emotions mediate the relationship between nursing students’ risk perceptions and professional commitment.

Psychological capital is a psychological state that can trigger positive behaviors in an individual’s growth and development process and comprises four psychological resources: self-efficacy, hope, optimism, and resilience (Luthans and Youssef, 2004). Psychological capital is a positive psychological resource that helps overcome problems (Luthans et al., 2007). As confirmed by a survey of Chinese doctors, psychological capital is a positive resource against depression and can moderate the effects of depression-related predictors on depression outcomes (Shen et al., 2014). Another recently published study by Park and Cho indicated that psychological capital was negatively correlated with COVID-19 risk perception (Park and Cho, 2022). According to the conservation of resources theory, individuals try to protect and maintain their existing resources and proactively acquire and establish new resources while avoiding resource loss (Hobfoll, 2011). A series of recently published studies reported that nursing students had better psychological capital during the COVID-19 pandemic and that their psychological capital was positively correlated with perceived stress (Wang et al., 2021**;** Sun et al., 2022). Based on these findings, it can be speculated that when negative emotions are generated along with emotional loss due to risk perception of the COVID-19 pandemic, individuals’ psychological systems produce a stress response to strive for more psychological capital to reduce negative emotions. Thus, Hypothesis 3 was proposed: psychological capital moderates the mediating effect of negative emotions.

**Fig. 1:**
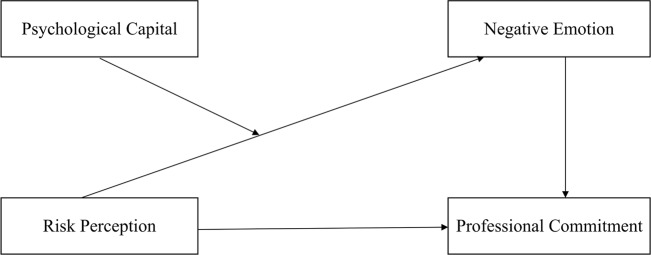
The hypothesis conceptual model. A description of the hypothesis relationship between variables in the study.

Based on the evidence previously identified in our literature review, a hypothetical conceptual model (Fig. 1) was developed to investigate the potential mechanism underlying the association between risk perception and professional commitment among nursing students in China, particularly in the current context of the COVID-19 pandemic.

## Methods

### Study design and participants

A descriptive cross-sectional design was used to examine nursing students from three colleges in Sichuan, China. Full-time junior college and undergraduate nursing students were included in this study. The study followed the STROBE guidelines, and all students voluntarily participated. A total of 1300 students were invited to participate in the online survey, and 1142 forms received from the participants were retained for analysis after screening for errors.

### Data collection instruments

The data collection tools used in this study included a general information questionnaire and risk perception, professional commitment, negative emotions, and psychological capital scales.

The researchers originally designed and developed the general information questionnaire to collect the demographic characteristics of nursing students, including sex, age, grade, whether they were the only child in the family, academic record, volunteer experiences during the COVID-19 pandemic, and experience as a student leader.

The risk perception scale was developed by Yan and Wen (Yan and Wen, 2020). The scale was used to assess the risk perception of nursing students and included two dimensions, personal and social risk perception, with a total of eight items. This scale uses the Likert 5-level scoring method, ranging from “totally disagree” to “totally agree,” which are assigned the value 1 and 5, respectively. All items scored positively, with higher scores indicating a higher perceived level of pandemic risk. In this study, Cronbach’s α of the scale was 0.803.

The professional commitment scale was compiled by Lian et al. (Lian et al., 2005). This scale is used to evaluate the professional commitment of nursing students, including emotional, ideal, normative, and continuous commitment, and contains 27 items. The scale uses the Likert 5-level scoring method, ranging from “completely unqualified” to “completely qualified,” which are assigned the values 1 and 5, respectively. The reverse-predefined items were scored oppositely. A higher score indicated a higher level of professional commitment. In this study, Cronbach’s *α* of the scale was 0.939.

The negative emotion scale was developed by Watson et al. and revised by Huang et al. (Huang et al., 2003**;** Watson et al., 1988). This scale was intended to evaluate nursing students’ negative emotions. The scale consists of ten items based on the adoption of the Likert 5-level scoring method, ranging from “almost none” to “extremely many,” which are assigned as 1 and 5, respectively. All items scored positively. Higher scores indicate higher levels of negative emotions. The Cronbach’s α of the negative emotion scale was 0.952.

The psychological capital scale was developed by Zhang et al. (Zhang et al., 2010). This scale was used to evaluate the psychological capital of nursing students and included four dimensions—self-efficacy, resilience, hope, and optimism—with a total of 26 items. This scale depends on adopting a seven-point Likert 7-level scoring method, in which reversely predefined items are associated with opposite scores. A higher score indicates a higher level of positive psychological capital. In this study, Cronbach’s α was 0.920.

### Data collection

Because of COVID-19 related health restrictions and protocols, this study did not conduct a field investigation. Instead, the Questionnaire Star website, an online survey platform, was used to collect data. Hyperlinks for accessing the questionnaire and QR codes were disseminated to nursing students at the three colleges for the investigation. Typical problems with online surveys include low response rates, low representativeness, and low item responses (Grande et al., 2022). To avoid these issues and improve the quality of the online survey, the following steps were taken. First, a detailed description section was added to the questionnaire to ensure that the respondents understood the study objectives, the necessity of collecting information from the survey, and the specific requirements for completing the survey forms to ensure the accuracy of the data. Informed consent was obtained from all participants before the survey. Second, the questionnaire used appropriate student language to describe questions and options, avoiding using grids or matrices to represent Likert-scale answers (Grande et al., 2022). Finally, after the survey, the questionnaire data were exported from the Questionnaire Star website for review and sorting to exclude incomplete forms and outliers.

### Data analysis

SPSS26.0 was used for the data processing and analysis. Statistical significance was set at *P* < 0.05 (two-tailed). General data were presented as frequencies and constituent ratios (%). The item scores on each scale were presented as mean (±standard deviation). Pearson’s correlation analysis was used to analyze correlations between variables. PROCESS Macro Models 4 and 7 were used to construct multiple regression models to evaluate mediation and moderation effects. The bootstrap 95% confidence interval (CI) of the model was calculated to determine the significance of the mediating and moderating effects. The mediation effect refers to the relationship between variables, X → Y is an indirect effect through intermediate variable M, and this indirect causal relationship is called the mediation effect (Baron and Kenny, 1986). The moderation effect refers to the moderating effect of variable *U* when the size or positive and negative directions of the correlation between variables *X* and *Y* are influenced by variable *U* (Baron and Kenny, 1986).

## Results

### Participant characteristics

The descriptive characteristics of the respondents (*n* = 1142) are presented in Table 1. The study group included 72 males (6.30%) and 1070 females (93.70%), with an average of (19.852 ± 1.284) years.

**Table 1:** The demographic characteristics of nursing students (*n* = 1142).

Variable	Category	*N* (%) or mean ± SD
Gender	Male	72 (6.30)
	Female	1070 (93.70)
Age (years old)		19.852 ± 1.284
School year	1st year	419 (36.69)
	2nd year	340 (29.77)
	3rd year	269 (23.56)
	4th year	114 (9.98)
Only child or not	Yes	274 (23.99)
	No	868 (76.01)
Academic record	Above	198 (17.34)
	Medium	594 (52.01)
	Lower	350 (30.65)
Volunteer experience during COVID-19 pandemic	Yes	297 (26.01)
	No	845 (73.99)
Experience as a student leader	Yes	291 (25.48)
	No	851 (74.52)

### Correlation analysis of risk perception, professional commitment, negative emotions, and psychological capital of nursing students

Table 2 shows the scores for each variable and the correlations between the variables investigated in this study. The scores of risk perception, negative emotions, psychological capital, and professional commitment of nursing students were (4.070 ± 0.612, range = 1–5), (2.608 ± 0.844, range = 1–5), (4.941 ± 0.700, range = 1–7), and (3.859 ± 0.501, range = 1–5), respectively. As indicated by the statistical results, nursing students’ risk perceptions were significantly and positively correlated with psychological capital (*r* = 0.267, *P* < 0.010), professional commitment (*r* = 0.249, *P* < 0.010), and negative emotions (*r* = 0.070, *P* < 0.050). Psychological capital was significantly positively correlated with professional commitment (*r* = 0.694, *P* < 0.010) but negatively correlated with negative emotions (*r* = −0.201, *P* < 0.010). Professional commitment was significantly and negatively correlated with negative emotions (*r* = −0.065, *P* < 0.050).

**Table 2:** Correlations between risk perception, psychological capital, professional commitment and negative emotions of nursing students.

Variables	*M* ± SD	Range	Risk perception	Psychological capital	Professional commitment	Negative emotions
Risk perception	4.070 ± 0.612	1–5	1			
Psychological capital	4.941 ± 0.700	1–7	0.267^**^	1		
Professional commitment	3.859 ± 0.501	1–5	0.249^**^	0.694^**^	1	
Negative emotions	2.608 ± 0.844	1–5	0.070^*^	−0.201^**^	−0.065^*^	1

^**^P < 0.01, ^*^P < 0.05.

### Analysis of the mediating effects of negative emotions

As shown in Table 3, nursing students’ risk perception was a significant predictor of professional commitment (*B* = 0.686, *P* < 0.001) and negative emotions (*B* = 0.121, *P* = 0.018). When both risk perception and negative emotions were included in the model, as shown by the bias-corrected bootstrap, the bootstrap 95% CI did not contain zero, verifying the mediating effect of negative emotions. Meanwhile, significant associations were still observed between risk perception and professional commitment (*B* = 0.702, *P* < 0.001) and between negative emotions and professional commitment (*B* = −0.133, *P* = 0.004), indicating that negative emotions partially mediated the relationship between risk perception and professional commitment. This mediation effect was −0.016 (95% CI [−0.037, −0.001]), which validated the hypothesis that negative emotions are an essential mediator in the relationship between risk perception and professional commitment in nursing students.

**Table 3:** The mediating effects of negative emotions.

Step	Variables	*B*	SE	*P*	95% CI		*R*^2^
					LLCI	ULCI	
1	*X* *→* *Y*	0.686	8.663	<0.001	0.531	0.842	0.062
2	*X* *→* *M*	0.121	0.051	0.018	0.021	0.221	0.005
3	*X* *→* *Y*	0.702	0.079	<0.001	0.547	0.858	0.069
	*M* *→* *Y*	−0.133	0.046	0.004	−0.223	−0.043	
Indirect effect	*X* *→* *M* *→* *Y*	Effect	Boot SE	Boot 95% CI			
		−0.016	0.009	−0.037 ~ −0.001			

*X* risk perception, *Y* professional commitment, *M* negative emotions, *CI* confidence interval, *LLCI* lower limit confidence interval, *ULCI* upper limit confidence interval.

### Analysis of the moderating effects of psychological capital

As shown in the first step of Table 4, risk perception had a significant direct effect on negative emotions (*B* = 0.246, *P* < 0.001), and the risk perception × psychological capital interaction effect on negative emotions was also significant (*B* = 0.007, *P* = 0.005). In Step 2, the direct effects of risk perception on professional commitment were significant (*B* = 0.702, *P* < 0.001), and the effects of negative emotions on professional commitment were also significant (*B* = −0.133, *P* = 0.004).

**Table 4:** The moderating effects of psychological capital.

Step	Independent variables	Dependent variables	*B*	SE	*P*	95% CI		R^2^
						LLCI	ULCI	
1	*X*	*M*	0.246	0.052	<0.001	0.144	0.347	0.064
	*W*		−0.114	0.014	<0.001	−0.141	−0.087	
	*X* *×* *W*		0.007	0.002	0.005	0.002	0.012	
2	*X*	*Y*	0.702	0.079	<0.001	0.547	0.858	0.069
	*M*		−0.133	0.046	0.004	−0.223	−0.043	
Conditional indirect effect	Psychological capital		Indirect effect	Boot SE	*P*	Boot LLCI	Boot ULCI	
	Mean − 1 SD		−0.016	0.012	>0.05	−0.044	0.003	
	Mean		−0.033	0.014	<0.05	−0.064	−0.008	
	Mean + 1 SD		−0.050	0.021	<0.05	−0.095	−0.014	
Moderated mediation Index = −0.001				0.001		−0.002	−0.0001	

*X* risk perception, *Y* professional commitment, *M* negative emotions, *W* psychological capital, *CI* confidence interval, *LLCI* lower limit confidence interval, *ULCI* upper limit confidence interval.

**Fig. 2:**
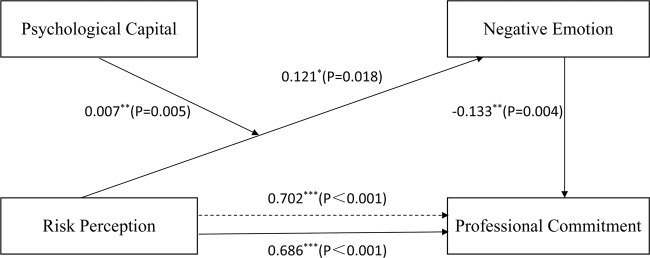
The moderated mediation model. The final model with path coefficients in the study.

The conditional indirect effects of psychological capital on the indirect path from risk perception through negative emotions to professional commitment were analyzed. Nursing students with higher psychological capital (+1 SD) revealed less increase in negative emotions induced by risk perception. In contrast, nursing students with lower psychological capital (–1 SD) showed a higher increase in negative emotions due to risk perception. The moderated mediation index was −0.001 (95% CI [−0.002, −0.0001]), indicating that the mediating effects of negative emotions were significantly moderated by psychological capital. These findings provide statistical evidence supporting our conceptual hypothesis. Figure 2 shows the final model with path coefficients.

## Discussion

In this study, the professional commitment score of nursing students was found to be (3.859 ± 0.501, range = 1–5), which was higher than the scale’s medium threshold value of 3 and the findings reported by relevant studies before the outbreak of COVID-19 in multiple countries, including China, Norway, Iran, and Italy (Nesje, 2015**;** Safari and Yoosefpour, 2018**;** Sollami et al., 2018**;** Zhuang et al., 2020). This finding suggests that the professional commitment of most Chinese nursing students was relatively high during the COVID-19 pandemic. Meanwhile, a positive correlation was found between nursing students’ risk perception and their professional commitment, which means that as nursing students’ perceived risk increased during the ongoing pandemic, their professional commitment was not compromised but instead further fortified and reflected stronger beliefs and motivations toward their future career paths. This seems contrary to Hypothesis 1 and different from Kaya’s view that higher risk will negatively impact the commitment of nursing professionals during the pandemic (Kaya et al., 2022). There are two possible explanations for the discrepancy. First, it should be noted that the COVID-19 pandemic has created an unprecedentedly challenging climate for worldwide healthcare systems, where nurses have gained great respect from various dimensions of society for their indispensable contributions to disease prevention and control (Liu et al., 2020). As a result, the social status of nurses has drastically improved compared to the pre-COVID-19 situation, when nursing is broadly recognized as a high-risk occupation with relatively low compensation and poor scope for career development, which is inconsistent with the high responsibilities that nurses shoulder while working in various clinical settings (Xu, 2008). The perception of the high risk of a pandemic also means higher returns, which will, to some extent, stimulate nursing students’ motivation to improve their professional commitment and strengthen their faith in dedicating themselves to their nursing occupations. Moreover, the medical expertize and clinical experience that nursing students gain from their academic training enable them to assess the level of risk associated with current progress in disease prevention and control from a more professional perspective. Therefore, the perception of high risk has great potential to boost nursing students’ desire to further improve their professional knowledge and skills and their beliefs about the value of their occupation by displaying more positive attitudes toward future career development. Second, such findings might have been induced in the particular context of China, thus presenting as inconsistent with findings from other nations, such as Spain, where 46.55% of nurses expressed their intention to discontinue their jobs (Santana López et al., 2021). In Canada, 29.5% of nurses showed a high willingness to leave the setting, and another 22.3% decided to quit their occupation (Lavoie-Tremblay et al., 2022). Finally, in South Korea, more than 10% of nurses contemplated quitting their jobs due to the risk of infection during the pandemic (Jang et al., 2021). In contrast, 96.8% of nurses in China spontaneously participated in pandemic prevention and control by showing an impressive willingness to work and professional commitment (Ke et al., 2021). This might be associated with the educational pattern of medical training programs in the context of Chinese culture, where medical professionals pursue the noble spirit of dedicating themselves to their lifetime careers in the healthcare industry, which prioritizes saving lives. Healthcare professionals have made irreplaceable contributions to the prevention and control of COVID-19 during the pandemic with remarkable altruism and professionalism and have gained unprecedented recognition and respect from society. They serve as role models and influence younger medical professionals (Bahmanbijari et al., 2017**;** Chen et al., 2021). In such a climate, nursing students’ confidence in the value of their occupation is significantly enhanced, further improving their self-recognition as nursing candidates and boosting their professional commitment level (Zhang and Li, 2020).

This study showed that nursing students’ risk perception was positively correlated with negative emotions. In contrast, negative emotions were negatively correlated with professional commitment and played a mediating role between risk perception and professional commitment during the pandemic. This validates Hypothesis 2, which implies that nursing students’ risk perception could positively impact their professional commitment but could also compromise such professional commitment by increasing negative emotions. In our study, direct effects dominated intermediary effects, resulting mainly in positive outcomes. Affected by the perception of pandemic-induced risk, conflicts were induced within nursing students’ mental status, which enabled them to have increased awareness of their occupational value and responsibilities as an indispensable group within society but also induced negative emotions that made them skeptical about their future career paths. Since the outbreak of COVID-19, pandemic-induced negative emotions have become a critical issue in China and among a wide range of population groups worldwide (Kim and Park, 2021). For example, negative emotions such as depression and anxiety among college students are constantly intensifying in Spain, Switzerland, and Bangladesh (Elmer et al., 2020**;** Fawaz and Samaha, 2021; Odriozola-González et al., 2020). In Spain, 21.34%, 34.19%, and 28.14% of college students reported moderate-to-extreme anxiety, depression, and stress, respectively (Odriozola-González et al., 2020). It is noteworthy that compared with students who specialize in non-medical majors, nursing students tend to have a higher level of negative emotions when confronted with public crises because of their professional attributes as well as heavy academic and internship workloads (Li et al., 2021**;** Savitsky et al., 2020). Negative emotions could be further induced, resulting in nursing students’ skepticism regarding the uncertainty of occupational safety with long-term exposure to high infection risk and whether they would have enough competence to handle challenging tasks as nurses (Kim and Park, 2021**;** Tang et al., 2020). Recent studies have identified undesired psychological disorders, such as psychological imbalance, tension, and anxiety, among nursing students because of the negative emotions induced by the pandemic. Undoubtedly, it will be difficult for nursing students to maintain their enthusiasm for learning under such psychological conditions, further weakening their professional commitment as healthcare providers (Ye et al., 2020**;** Zhao et al., 2021).

Regarding Hypothesis 3, our study confirmed that psychological capital moderates the mediating effect of negative emotions. Specifically, nursing students with high psychological capital experienced fewer negative emotions induced by risk perception than those with low psychological capital. In other words, nursing students’ psychological capital can buffer the negative emotions caused by risk perception. Psychological capital is a positive state in which individuals store energy inward (Luthans and Youssef, 2004), which functions as an energy supplement and motivation stimulation (Schaufeli et al., 2002). The self-depletion theory highlights that individual psychological resources are limited (Baumeister et al., 1998). Therefore, individuals mobilize their internal resources in response to perceived risk. However, if such internal resources are not supplemented on time, the retrieval process will be blocked after depletion, eventually damaging mental health. High psychological capital is closely related to positive emotions (Avey et al., 2008), and individuals with higher psychological capital have more positive resources to deal with risks (Xiong et al., 2020). Therefore, in the context of COVID-19, nursing students should stimulate their psychological capital to mobilize their resources to deal with external risks. The higher the loss of resources supplemented by nursing students with higher psychological capital, the fewer negative emotions they will have, with a lower impact on professional commitment. Although the moderating effect of psychological capital was significant in our study, it was small, indicating that its role could also be affected by other factors. Nevertheless, as an important internal protective factor, psychological capital still positively contributes to the mental health of nursing students during the COVID-19 pandemic.

## Limitations and recommendations

This study had several limitations. First, as part of a cross-sectional study, data were collected at a particular time point during the COVID-19 pandemic, which failed to reflect changes in all the variables investigated over time. Second, convenience sampling enabled the collection of data for analysis from three colleges in Sichuan Province, thus compromising the representativeness of the study sample to a certain extent. Third, because the data analyzed in this study were collected using a self-reported approach, the accuracy of the data may have been affected by social desirability. Fourth, although the moderating and mediating variables were significant, the correlations were weak in some cases, which may have reduced the robustness of the conclusions. In future studies, strategies such as expanding the research scope or using random sampling methods could be adopted to improve the representativeness of this study. Longitudinal studies can be used to further examine the causal relationships between variables, and incorporating multiple data collection methods during the data collection process can improve the reliability and validity of the study.

## Conclusion

This is the first study to explore the impact of nursing students’ risk perceptions on their professional commitment to China during the ongoing COVID-19 pandemic. Specifically, we studied the relationship between risk perception and professional commitment in nursing students as well as the roles played by negative emotions and psychological capital throughout this process. Our findings indicate that nursing students’ risk perception directly and positively affects their professional commitment, and negative emotions mediate between risk perception and professional commitment. Psychological capital also moderated the mediating effect of negative emotions. To ensure the healthy and sustainable development of nursing professionals during the COVID-19 pandemic, effective intervention strategies are urgently needed to promote nursing students’ professional commitment levels by mitigating negative emotions and improving their psychological capital. In addition to academic institutions that bear the major responsibility of cultivating highly skilled nursing professionals, other functional divisions of society should also have adequate awareness of this critical problem to proactively engage in the implementation of supportive strategies in this challenging climate.

## Implication for nursing education and practice

Based on our findings, the following suggestions are proposed to inform the implementation of interventions currently needed from multiple perspectives:

From an educational perspective, multiple aspects associated with nursing students’ professional commitment should be addressed as essential components of their academic training programs, including professionalism cultivation, occupational competence enhancement, and mental health promotion. As such, the quality and teaching patterns of medical-related courses, such as public health and humanistic and psychological nursing, should be further optimized to improve nursing students’ capacity to deal with public emergencies, as well as to ensure that they have strong professional beliefs and an in-depth understanding of medical expertize when confronted with real-world challenges. Additionally, schools and internship medical institutes must establish a surveillance system to manage nursing students’ behaviors and practices during public health emergencies. The provision of risk-targeted training courses is also necessary to enhance nursing students’ coping skills in response to crises, such as risk management and control, and to apply risk-specific medical expertize and clinical practices to occupational safety in various clinical settings. Mental health education is undoubtedly another essential intervention that alleviates nursing students’ negative emotions induced by risk perception, along with other risk-targeted psychological interventions and the provision of mental health counseling services, both on campus and in clinical settings where students are working towards their internship goals.

From the perspective of the public and society, it is essential to provide nurses with sufficient trust, tolerance, support, and praise to create a friendly environment for healthcare providers in which their selfless contributions throughout the public health crisis are recognized and respected. This would significantly improve nursing candidates’ confidence in the value of their occupation, which would further mitigate uncertainties about future career development and enhance their professional commitment. Because occupational safety concerns for healthcare providers during the pandemic are exacerbated by the lack of protective facilities, efforts should be made at the government level to mitigate concerns regarding material supply. This could alleviate nursing students’ concerns about the safety of working on disease prevention and control.

At the individual level, nursing students must improve their competencies to be better prepared for the challenges embedded in their future career paths. In addition to gaining an in-depth understanding of medical expertize through academic training, students are expected to acquire practical knowledge in disease prevention and control to handle various tasks during public health crises professionally. As pandemic-induced anxiety and uncertainty would inevitably affect nursing students’ mental health, activities to release pressure are also highly recommended, such as sticking to healthy workout habits, listening to soothing music, trying to alleviate stress by focusing on positive aspects and inspiring news reported by the mainstream media, or seeking mental counseling services promptly if those pressures cannot be effectively handled. These strategies are expected to improve students’ psychological capital and reduce the impact of negative emotions on their professional commitment.

## Data Availability

The datasets generated during and/or analyzed during the current study are available from the corresponding author on reasonable request.
